# Contrasting clinical outcomes in two cohorts of cats naturally infected with feline immunodeficiency virus (FIV)

**DOI:** 10.1016/j.vetmic.2014.12.023

**Published:** 2015-03-23

**Authors:** Paweł M. Bęczkowski, Annette Litster, Tsang Long Lin, Dominic J. Mellor, Brian J. Willett, Margaret J. Hosie

**Affiliations:** a MRC Centre for Virus Research, University of Glasgow, Glasgow, UK; b Small Animal Hospital, University of Glasgow, Glasgow, UK; c Department of Veterinary Clinical Sciences, Purdue University, West Lafayette, IN 47907, USA; d Indiana Animal Disease Diagnostic Laboratory and Department of Comparative Pathobiology, Purdue University, West Lafayette, IN 47907, USA; e School of Veterinary Medicine, University of Glasgow, Glasgow, UK

**Keywords:** FIV, Natural infection, Clinical outcome, FIV load, CD4:CD8, Lymphoma

## Abstract

Despite over 25 years of feline immunodeficiency virus (FIV) research, relatively little is known about the longitudinal course of FIV infection following natural infection. In contrast to published reports of experimental infections using lethal strains of the virus, clinical signs of naturally acquired FIV infection can be mild or inapparent, rather than life-threatening. In this prospective, longitudinal controlled study, based in Chicago, IL (*n* = 17) and Memphis, TN (*n* = 27), we investigated two cohorts of privately owned, naturally infected cats kept under different housing conditions. Cats in the Chicago cohort (Group 1) were kept in households of ≤2 cats, while the Memphis cohort (Group 2) comprised part of a large multi-cat household of over 60 cats kept indoors only, with unrestricted access to one another.

The majority of cats from Group 1 did not display clinical signs consistent with immunodeficiency during the 22-month observation period. In contrast, the outcome of infection in Group 2 was dramatically different; 17/27 (63%) of cats lost a median of 51.3% of their bodyweight (*P* < 0.0005) and died during the study period, with lymphoma being the most common cause of mortality.

Although the decrease in CD4+ T cell count between enrolment and terminal disease was significant (*P* = 0.0017), the CD4:CD8 ratio at the time of enrolment did not reliably distinguish FIV-positive cats classified as ‘healthy’ and ‘not healthy’ at either cohort. FIV load at enrolment was significantly lower in Group 1 than in Group 2 (*P* < 0.0001), but there were no significant differences at enrolment between healthy and not healthy cats at either group.

In conclusion, the results of this study suggest that management and housing conditions impact on disease progression and survival times of FIV-positive cats.

## Highlights

• Multi-cat household animals displayed 63% mortality rate.
• Lymphoma was the most common cause of death.
• The CD4:CD8 ratio failed to distinguish cats classified as healthy and not healthy.
• FIV load failed to distinguish cats classified as healthy and not healthy.
• Management and housing conditions impact on the progression of FIV infection.

## 1 Introduction

Feline immunodeficiency virus (FIV) is an important pathogen of domestic cats that is distributed worldwide. Prevalence estimates vary from 1% to 44%, depending on the geographical location as well as the entry criteria of each study (Hartmann, 1998; Ishida et al., 1989). Outdoor, adult, male, sick cats with bite wounds are at increased risk of infection, since transmission is mainly via bites from infected cats (Hartmann, 1998). Clinical signs are similar to those described for human immunodeficiency virus-acquired immune deficiency syndrome (HIV-AIDS) and include signs of immunosuppression, hematopoietic changes and neoplasia (Hartmann, 2011). The progression of experimental FIV infection can be monitored using the staging system used in HIV infection: acute, persistent generalized lymphadenopathy, asymptomatic carrier phase (AC), AIDS-related complex (ARC) and AIDS (English et al., 1994). While this classification is useful in well-controlled, experimental settings, it has limitations under field conditions, mainly because of strain-related variability in pathogenicity, variation in the ages of cats at the time of infection as well as possible exposure to secondary pathogens (Hartmann, 2012; Pedersen et al., 2001; Podell et al., 1997). Hence, there have been variable morbidity and mortality rates reported in naturally infected cats (Addie et al., 2000; Hofmann-Lehmann et al., 1997; Kohmoto et al., 1998; Liem et al., 2013).

Providing an accurate prognosis for FIV-positive cats is important so that optimal care can be provided by veterinarians and cat owners. Surrogate markers for HIV infection are well established and valuable in monitoring both responses to therapy and the likelihood of disease progression (Churchill, 1997; Katzenstein, 2003; Okonji et al., 2012; Shaunak and Teo, 2003; Smith and Stein, 2002). In contrast, to our knowledge, prospective controlled studies investigating the course of FIV infection and markers of disease progression have not been described. Consequently, the longitudinal course of naturally acquired FIV infection is poorly described and the variability in rates of progression to immunodeficiency are poorly understood.

In this controlled study, we examined clinical and laboratory parameters from two cohorts of cats living in different locations and kept under different housing conditions. The aim of the study was to report clinical findings, post-mortem findings, bodyweight, CD4+ T cell counts, FIV load and phylogenetic classification of viral envelope genes in two groups of cats naturally infected with FIV.

## 2 Materials and methods

### 2.1 Cats

Forty-four neutered, FIV-positive (SNAP FIV/FeLV Combo Test (IDEXX Laboratories, Westbrook, MN) cats were enrolled, with an equal number of age- and sex-matched FIV-negative cats from the same geographical locations. In order to obtain an adequate sample size FIV-positive cats and matched control cats during the 2-year enrolment period, it was necessary to source cats from two separate locations. FIV-positive results were further confirmed by virus isolation (Hosie et al., 2009). All cats were feline leukaemia virus (FeLV) antigen negative at enrolment. The number of cats enrolled into the study was capped at 44 due to the capacity of the laboratory at which molecular analyses were performed.

Seventeen of the 44 FIV-positive cats enrolled (Group 1) had been previously adopted from a large metropolitan adoption-guarantee shelter (PAWS Chicago) and lived in single-cat households in Chicago, IL, USA except for seven cats: two cats (C7 and C4) cohabited in a two-cat household; one cat (C13) lived in a two-cat household with a FIV-negative cat enrolled in the study; one cat (C9) was housed at PAWS Chicago for the first 11 weeks of the study and then was adopted into a house with an FIV-positive cat not enrolled in the study; and three cats (C2, C15 and C21) were housed at PAWS Chicago in a room containing up to three FIV-positive cats before they were each adopted into single cat households at 2, 14 and 58 weeks after enrolment, respectively.

The remaining twenty-seven FIV-positive cats enrolled (Group 2) were housed together in a large multi-cat household operating as an FIV-positive cat rescue in Memphis, TN, where a total of 53 FIV-positive and 10 FIV-negative cats were housed indoors with unrestricted access to one another. None of 10 FIV-negative cats from the Memphis cat rescue died during the study period. Since these cats were not age- or sex-matched for any of the enrolled FIV-infected cats from the same household (Group 2), their health status was not monitored using the study protocol. All enrolled FIV-negative cats at both locations lived in households of three cats or fewer.

The study and its aims were reviewed and approved by University of Glasgow Ethics Committee and the Purdue Animal Care and Use Committee. Cat owners provided written informed consent for their participation in the study.

### 2.2 Study timeline and collection dates

At enrolment, all cats underwent a general physical examination by a registered specialist in feline medicine (AL) and blood was collected for determination of FIV status and laboratory analysis. Oral examinations were conducted as part of the physical examination and a gingival score (0–3/3; Lobprise, 2007) was assigned. At the time of enrolment, FIV-positive cats were classified as ‘healthy’ if there were no abnormalities on physical examination and their gingival score was 0/3 or 1/3. Cats were classified as ‘not healthy’ if at least one clinical abnormality was detected, or if their gingival score was 2/3 or 3/3. The clinical classification at enrolment was based solely on physical examination findings and remained with each cat for the 22-month study period, regardless of any further changes. An entry criterion for age- and sex-matched FIV-negative cats was healthy status at the enrolment examination.

Serial physical examinations and specimen collections were made at 6-monthly intervals in FIV-positive cats over the study period. Physical examinations and specimen collections were performed at enrolment and 12 months later in FIV-negative cats. The date of the first known positive FIV ELISA test was obtained for all FIV-positive cats and the time between first diagnosis and study enrolment (months) was calculated. Abnormalities detected during the clinical examinations are listed in Table 1.

### 2.3 Laboratory and post-mortem examinations

Flow cytometric analyses were performed as previously described (Lin and Litster, 2013). FIV loads were measured using a commercially available PCR test (IDEXX FIV RealPCR Test, IDEXX Laboratories, West Sacramento, CA). The assay detects the presence of viral nucleic acid, including both genomic DNA and viral RNA, in peripheral blood leukocytes with 80.5% sensitivity and 99.9% specificity (IDEXX Laboratories, West Sacramento, CA).

Post-mortem examinations were performed by a specialist veterinary pathologist (TLL).

### 2.4 Phylogenetic analysis

Maximum likelihood (ML) phylogenetic trees were constructed in MEGA5 (Tamura et al., 2011) under the HKY nucleotide substitution model, selected through jMODELTEST (Posada, 2008). Statistical support for the ML trees was estimated using 1000 bootstrap replicates (Efron et al., 1996). Multiple *env* sequences (*n* = 355), from cats enrolled in the study were subjected to rigorous recombination testing as described previously (Bęczkowski et al., 2014).

### 2.5 Statistical analysis

Graphing and statistical data analyses were performed using commercially available software (GraphPad Prism version 5.00, GraphPad Software). Descriptive data were shown as medians and interquartile range (IQR; median, 5th and 95th quartiles). Given the relatively small sample size, and after inspection of the data distributions, Mann–Whitney and Wilcoxon matched pairs tests were used to test hypotheses regarding differences in laboratory parameters between and within cat groups. Binary data were analyzed using Fisher's exact test. FIV load data were tested for correlation using Spearman correlation tests. Kaplan–Meier curves were compared using the Mantel–Cox ‘log-rank’ test and tested with the log-rank test for trends. Significance was set at *P* < 0.05.

## 3 Results

### 3.1 Cats

Table 2 provides breed, gender, age and time since first FIV-positive test data for all FIV-positive cats and for cats from Group 1 and Group 2. Each matching pair of FIV-positive and FIV-negative cats was aged within 2 years of each other. All enrolled cats remained FeLV-negative over the study period and none of the FIV-negative cats seroconverted.

### 3.2 Health status

In the FIV-positive group at the time of enrolment, there were equal numbers of healthy (*n* = 22) and not healthy animals (*n* = 22). In Group 1, 10 cats were classified as healthy (59%) and seven cats were classified as not healthy (41%); all but one cat remained in this classification during the observation period. In Group 2, 12 cats were initially classified as healthy (44%) and 15 cats that were classified as not healthy (56%) remained in these classifications throughout the study. However, 63% of cats (17/27) from Group 2 experienced severe weight loss and died during the study period. Half (6/12) of the cats classified as healthy and 73% (11/15) of cats classified as not healthy died during the study (*P* = 0.26). Almost half of cats diagnosed at post-mortem with lymphoma (44%; 4/9) had been classified as healthy at the time of enrolment (Table 3).

### 3.3 Post-mortem findings

**Fig. 1:**
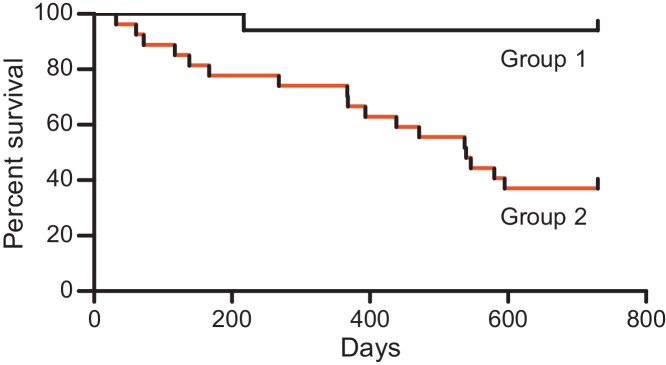
Kaplan–Meier survival plots for FIV-positive cats from Group 1 (black; *n* = 17) and Group 2 (red; *n* = 27). Survival rates in Group 1 and Group 2 over the 22-month study period were 94% and 37%, respectively (*P* = 0.0006, Gehan–Breslow–Wilcoxon test). (For interpretation of the references to colour in this figure legend, the reader is referred to the web version of this article.)

During the 22-month study period, 1/17 (5.9%) FIV-positive cats from Group 1 and 17/27 (63%) FIV-positive cats from Group 2 died. Fig. 1 illustrates a Kaplan–Meier survival plot of both cohorts. The cats that died were examined post-mortem and the findings are shown in Table 3. In Group 2, post-mortem data were not available for one cat (M33).

The most common pathological finding at post-mortem in Group 2 was lymphoma; various anatomical types were documented in 9/16 (56%) cats from Group 2 (Table 4). Four cats had lymphoma limited to a single site (retro-bulbar site in one case and bone marrow in three cases), while in five other cats, varying degrees of dissemination were observed. Bone marrow involvement was noted in all but one case. The second most common finding was nodal lymphoma, diagnosed in 5/9 cats (56%) and accompanied by splenic lymphoma in 3/9 cases (33%). One cat (M11) had a disseminated form involving the kidneys, liver, jejunum, heart, trachea and tongue. The median time since first FIV-positive ELISA test to death in cats with lymphoma was 5.3 years (range, 2.9–9 years).

Lymphoid hyperplasia and lymphoid depletion were noted concurrently in 13/16 (81%) cats. These findings were documented in various tissues, mostly in the spleen and lymph nodes, but in four cats, Peyer's patch atrophy was also diagnosed. Erythroid bone marrow hyperplasia was documented in 9/16 cats (56%), as was myeloid bone marrow hyperplasia. Multifocal lymphocytic interstitial nephritis was diagnosed in six cats (37.5%). In four cats, there were pathological signs of respiratory tract infection and pneumonia. Hypertrophic cardiomyopathy and signs of congestive heart failure were identified in the single FIV-positive cat from Group 1 that died.

None of the FIV-negative cats died during the study period.

### 3.4 Bodyweight

**Fig. 2:**
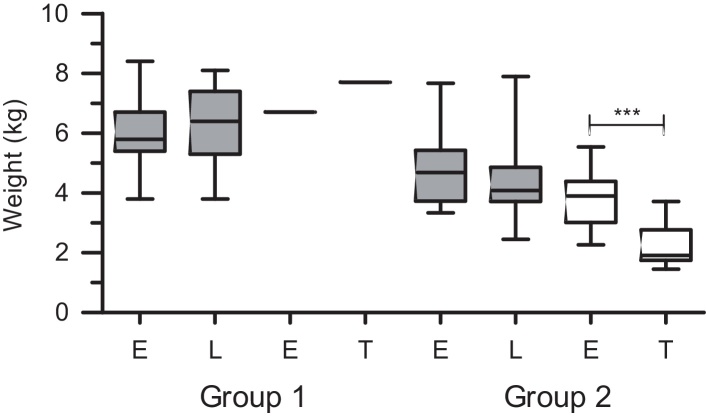
Bodyweights of FIV-positive cats from Group 1 (*n* = 17) and Group 2 (*n* = 27). E, enrolment weight; L, weight at the end of the study period; T, terminal weight. Grey boxes denote cats alive at the end of the 22-month study period; White boxes denote cats dead at the end of the 22-month study period. (The central bar in the box denotes the median, while the top and bottom of the box represent the 75th and 25th centiles, respectively. The upper and lower bars represent the 95th and 5th centiles, respectively. ***P < 0.0005.)

The bodyweight at the time of death for the single FIV-positive cat from Group 1 cohort that died was 15% heavier than the bodyweight recorded at enrolment (Fig. 2). The 17 FIV-positive cats from Group 2 that died lost a median of 51.3% of their bodyweight between enrolment (median 3.9 kg, range 2.27–5.54 kg) and death (median 1.9 kg, range 1.45–3.72 kg; *P* < 0.0005), over a median time span of 15 months (range 1.6–20 months). Those cats lost a median of 10.9% of their bodyweight between enrolment and 3 months before death (range −26.3% to +9.3%); median bodyweight loss in the last 3 months of life was 29.5% (range −55.4% to −8.9%).

The 16 FIV-positive cats from Group 1 that survived for the entire study period gained a median of 7.6% bodyweight over the 12 months following enrolment (enrolment, median 5.9 kg, range 3.8–8.4 kg; 12 months later 6.35 kg, range 3.9–8.1 kg; *P* = 0.2089). In contrast, the 10 FIV positive cats from Group 2 that remained alive lost a median of 12.8% of their bodyweight over the 12 months following enrolment (enrolment, median 4.68 kg, range 3.34–7.67 kg; 12 months later, median 4.08 kg, range 2.45–7.89 kg; *P* = 0.2324).

FIV-negative cats from Group 1 for which bodyweight data were available (*n* = 16) gained a median 16.8% bodyweight between enrolment (median 5.35 kg, range 4–8.5 kg) and 12 months later (median 6.25 kg, range 4.1–8 kg; *P* = 0.0145). Similarly, FIV negative cats from Memphis for which bodyweight data were available (*n* = 24) gained a median of 24.5% bodyweight between enrolment (median, 4.9 kg, range 2.7–9 kg) and 12 months later (median 6.1 kg, range 2.6–9.9 kg; *P* = 0.0022).

Available bodyweight data from Group 2 demonstrated that enrolment weights for FIV-positive cats from Group 2 that died were significantly lower than those for matched FIV-negative cats (*n* = 17; FIV-positive, median 3.9 kg, range 2.27–5.54; FIV-negative, median 5.6 kg, range 2.7–6.8 kg; *P* = 0.0046). Bodyweights were not statistically different when FIV-positive survivors from Group 2 and matching FIV-negative cats were compared at enrolment (*n* = 10; FIV-positive, median 5.12 kg, range 3.34–7.68 kg; FIV-negative median 5.8 kg, range 4.6–9.0 kg; *P* = 0.1641) and 12 months later (FIV-positive, median 4.63 kg, range 3.63–8.27 kg; FIV-negative, median 6.2 kg, range 4.9–9.9 kg; *P* = 0.0977).

### 3.5 CD4+ T cell counts and CD4:CD8 ratio

**Fig. 3:**
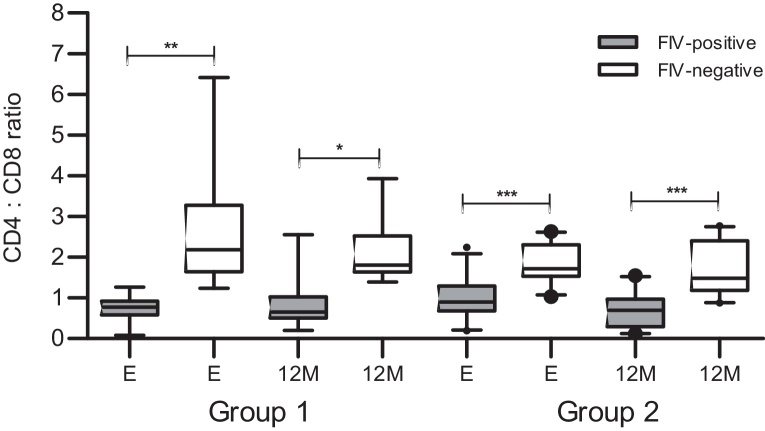
CD4:CD8 ratios for FIV-positive cats from Group 1 and Group 2 (in grey) and age- and sex-matched FIV-negative cats from the same geographic location (in white). For Group 1, data represent 17 FIV-positive cats and 17 age- and sex-matched FIV-negative cats at enrolment (E) and 16 surviving FIV-positive and 16 FIV-negative cats at 12 months after enrolment (12M). In Group 2 at enrolment, data were collected from 27 FIV-positive cats and 27 FIV-negative cats; 12 months after enrolment, data were collected from 21 surviving FIV-positive cats and 21 FIV-negative cats. (The central bar in the box denotes the median, while the top and bottom of the box represent the 75th and 25th centiles, respectively. The upper and lower bars represent the 95th and 5th centiles, respectively. *P < 0.01; **P < 0.001; ***P < 0.0001.)

Fig. 3 illustrates the CD4:CD8 ratios obtained over 12 months for the FIV-positive cats from Group 2 with reference values acquired from FIV-negative cats classified as healthy from the same geographic location. The difference between FIV-positive (median 0.89, range 0.19–2.24) and FIV-negative cats (median 1.71, range 1.03–2.62) was statistically significant at the time of enrolment (*P* < 0.0001) and 12 months later (medians 0.69, range 0.11–1.54 and 1.48, range 0.88–2.77 for FIV-positive and FIV-negative cats, respectively; *P* < 0.0001). The CD4:CD8 ratio in surviving FIV-positive cats was maintained over 18 months at a consistently low level (median values: 0.89, 0.73, 0.69, and 0.81 for each time point).

CD4:CD8 ratio at the time of enrolment (*P* < 0.0005) was lower in the FIV-positive cats from Group 1 (median 0.77, range 0.08–1.27) than in matched FIV-negative cats (median 2.18, range 1.23–6.42; Fig. 3). CD4:CD8 ratios in the FIV-positive cats from Group 1 were maintained at a consistently low level over the 12 months following enrolment (median 0.65, range 0.2–2.55) compared to age- and sex-matched FIV-negative cats (median 1.8, range 1.4–3.93; *P* = 0.0054). The CD4:CD8 ratios at the time of enrolment in the FIV-positive cats from Group 1 were lower (median 0.77, range 0.08–1.27) than those of the FIV-positive cats from Group 2 (median 0.89, range 0.19–2.23; Fig. 3).

**Fig. 4:**
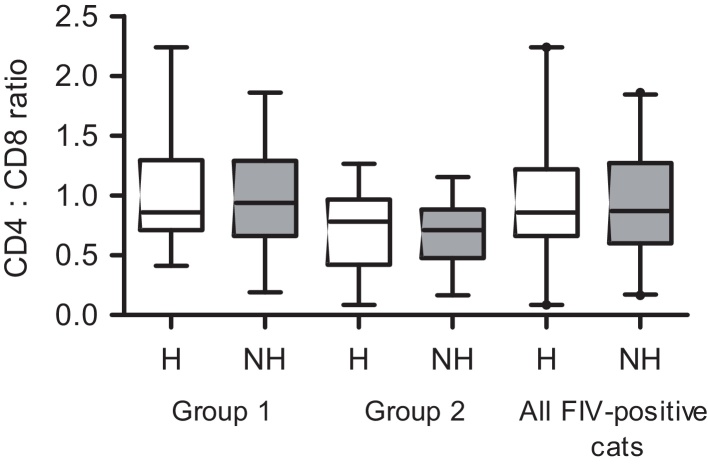
CD4:CD8 ratio at enrolment for FIV-positive cats from Group 1 (*n* = 17), Group 2 (*n* = 27) and all FIV-positive cats (*n* = 44). Cats were classified as healthy (H) or not healthy (NH) based on physical examination findings at the time of enrolment. (The central bar in the box denotes the median, while the top and bottom of the box represent the 75th and 25th centiles, respectively. The upper and lower bars represent the 95th and 5th centiles, respectively.)

There were no statistically significant differences when the CD4:CD8 ratio was compared between cats classified as healthy and not healthy at enrolment at either group, or for all FIV-positive cats as a single group (Group 1, *P* = 0.97; Group 2, *P* = 0.84; all FIV-positive cats, *P* = 0.99; Fig. 4).

**Fig. 5:**
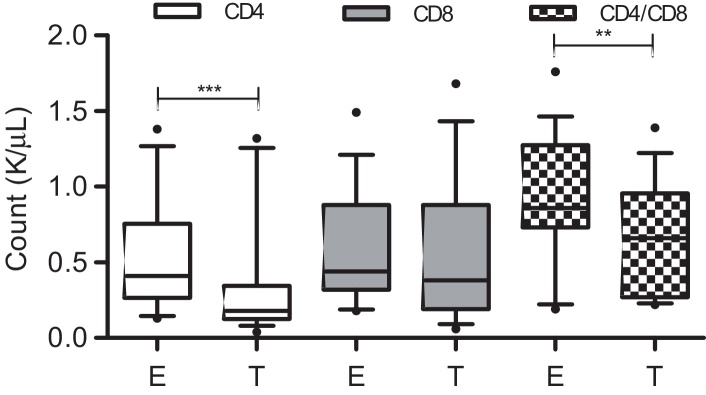
CD4+ and CD8+ T cell count and CD4:CD8 ratio at enrolment (E) and terminally (T) from the FIV-positive cats from Group 2 that died during the study (*n* = 17). (The central bar in the box denotes the median, while the top and bottom of the box represent the 75th and 25th centiles, respectively. The upper and lower bars represent the 95th and 5th centiles, respectively. **P = 0.0034; ***P = 0.0017.)

To determine the kinetics of the CD4:CD8 ratio inversion over the course of infection, we compared absolute CD4+ and CD8+ T lymphocyte numbers from 17 cats from Group 2 that died during the study (Fig. 5). The decrease in CD4+ T cell count was statistically significant when enrolment results (median 0.41 K/μL, range 0.13–1.38 K/μL) were compared with terminal results (median 0.18 K/μL, range 0.04–1.32 K/μL; *P* = 0.0017), but this was not the case for the decrease in CD8+ T cell count from enrolment (median 0.44 K/μL, range 0.18–1.49 K/μL) to terminal disease (median 0.38 K/μL, range 0.06–1.68 K/μL; *P* = 0.35). Therefore, the decrease in the CD4:CD8 ratio was attributed to a reduction in the CD4+ T cell count. Further analysis of total lymphocyte counts in deceased cats showed that the decrease in absolute lymphocyte numbers between enrolment (median 1.73 K/μL, range 1.15–4.03 K/μL) and terminal disease (median 1.25 K/μL, range 0.25–3.55 K/μL) reflected not only the loss of CD4+ T cells, but also depletion of CD21+ B cells (enrolment, median CD21+ B cell count 0.26 K/μL, range 0.12–0.89 K/μL; terminal specimens, median CD21+ B cell count 0.17 K/μL, range 0.04–0.37 K/μL; *P* = 0.0061).

### 3.6 FIV load (FIV genomes/mL blood)

**Fig. 6:**
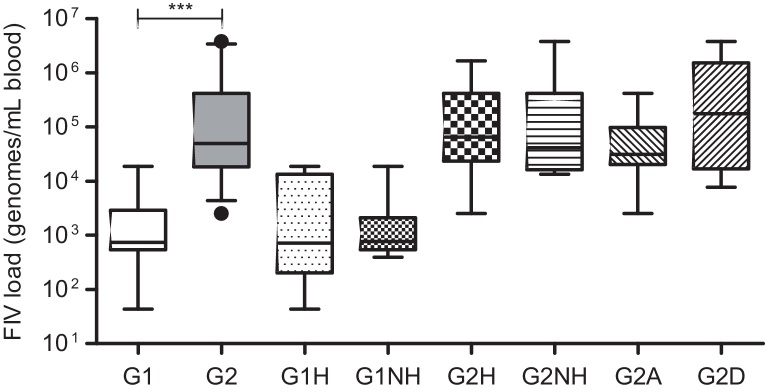
FIV load at the time of enrolment (genomes/mL blood). Statistical comparisons were made between pairs of classifications across the graph. G1, Group 1 (*n* = 15); G2, Group 2 (*n* = 26); H, healthy on the day of enrolment (no abnormalities detected on a physical examination; Group 1, *n* = 8, Group 2, *n* = 11); NH, not healthy on the day of enrolment (at least one abnormality detected on a physical examination; Group 1, *n* = 7, Group 2, *n* = 15); A, alive at the end of the 22-month study period (Group 2, *n* = 9); D, cats dead at the end of the 22-month study period (Group 2, *n* = 17). ***P < 0.0001.

The FIV load at enrolment was significantly lower in Group 1 (median, 741 genomes/mL blood; range 43–18,796 genomes/mL blood) than Group 2 (median, 50,003 genomes/mL blood; range, 2540–3,792,000 genomes/mL blood; *P* < 0.0001; Fig. 6). There were no statistically significant differences in FIV load at enrolment when healthy and not healthy cats were compared in Group 1 (healthy, *n* = 8, median 717 genomes/mL blood; range, 43–18,796 genomes/mL blood; not healthy, *n* = 7, median 767 genomes/mL blood; range, 395–18,796 genomes/mL blood; *P* > 0.05) or Group 2 (healthy, *n* = 11, median 64,843 genomes/mL blood; range, 2540–1,667,000 genomes/mL blood; not healthy, *n* = 15, median 41,387 genomes/mL blood; range, 13,423–3,792,000 genomes/mL blood; *P* > 0.05). In Group 2, there were no significant differences in FIV load at enrolment when the following comparisons were made: cats still alive vs. cats that had died by the end of the study period (alive, *n* = 9, median 31,395 genomes/mL blood; range, 2540–41,8671 genomes/mL blood; dead, *n* = 17, median 176,551 genomes/mL blood; range, 7724–3,792,000 genomes/mL blood; *P* > 0.05); cats that had died by the end of the study period and were classified at enrolment as either healthy or not healthy (healthy, *n* = 6, median 241,757 genomes/mL blood, range, 7724–1,667,000 genomes/mL blood; not healthy, *n* = 11, median 176,551 genomes/mL blood; range, 13,423–3,792,000 genomes/mL blood; *P* > 0.05); and cats that were still alive by the end of the study period and were classified at enrolment as either healthy or not healthy (healthy, *n* = 5, median 56,867 genomes/mL blood, range, 2540–418,671 genomes/mL blood; not healthy, *n* = 4, median 27,771 genomes/mL blood, range 16,176–43,138 genomes/mL blood; *P* > 0.05).

**Fig. 7:**
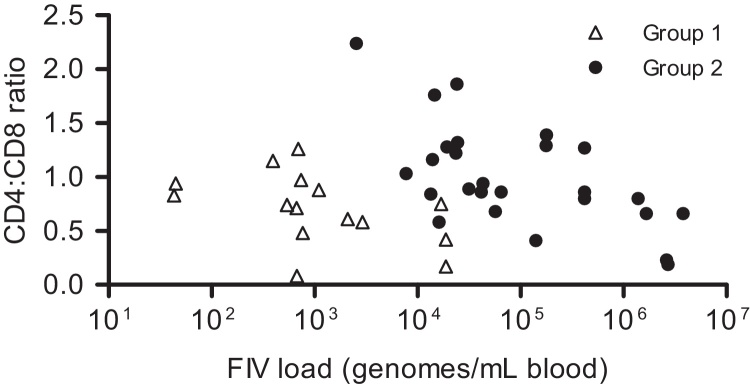
Correlation between FIV load (FIV genomes/mL blood) and CD4:CD8 ratio for FIV-positive cats from Group 1 (*n* = 15; *r* = −0.5022, *P* = 0.0564) and Group 2 (*n* = 26; *r* = −0.5479, *P* = 0.0038).

Statistical correlations between FIV load and CD4+ T cells, CD8+ T cells and the CD4:CD8 ratio were investigated for each group. There were statistically significant, or close to significant, negative correlations between the CD4:CD8 ratio and FIV load in Group 1 (*r* = −0.5022; *P* = 0.0564) and in Group 2 (*r* = −0.5479; *P* = 0.0038; Fig. 7).

### 3.7 Phylogenetic classification of FIV envelope genes

Full-length viral envelope (*env*) gene sequences from FIV-positive cats were examined (*n* = 355). In Group 1, clade B *env* viruses and clade A/B recombinant *env* viruses each accounted for 50% of infections. In Group 2, 69% of cats were infected with viruses bearing clade B *env*, while 31% of cats were infected with recombinant *env* viruses (Supplementary Table 3).

## 4 Discussion

Here we describe contrasting clinical outcomes of naturally acquired FIV infection, demonstrating significant differences between cats enrolled from two separate cohorts. The relationship between FIV infection and clinical disease is unclear, with some studies reporting that infected cats are at increased risk of morbidity and mortality (Muirden, 2002), while others report no such association (Akhtardanesh et al., 2010; Liem et al., 2013). Considering all 44 FIV-positive cats, there was no relationship between mortality and morbidity and FIV infection; however, this was not the case when the two cohorts were examined separately. Our observations in Group 2 indicated that living conditions might have played a role in the more rapid disease onset. In contrast, cats from Group 1 were generally housed in single cat households and remained alive and in relatively good health over the study period. Therefore, it appears that FIV infection is more likely to progress in cats kept in crowded shelter conditions compared to those living in spacious environments. As a corollary, our results suggest that the FIV-positive cats in our study that lived in single or dual cat households remained free of clinical signs of illness over the study period; this can most likely be attributed to lower risks of exposure to opportunistic pathogens and reduced levels of environmental stress.

FIV infected cats are at greater risk of opportunistic infections and neoplasia (Hartmann, 2012). Regular assessment of their health status is essential and, according to European Advisory Board on Cat Diseases guidelines (Hosie et al., 2009), health checks are recommended every 6 months. The short time to disease progression leading to the deaths of 63% of cats in Group 2 within the 22-month follow-up period was unexpectedly high, especially in light of previously published studies that were unable to demonstrate a difference in lifespan between FIV-positive and FIV-negative cats (Addie et al., 2000; Ravi et al., 2010). The health status of cats from Group 1 was similar to that of cats in a household in which FIV infection did not reduce life expectancy over a period of 2 years (Addie et al., 2000). There are several clinical abnormalities associated with FIV infection, with gingivostomatitis being the most common. Although one study (Quimby et al., 2008) did not observe an association between FIV infection and gingivostomatitis, the high prevalence observed here was in agreement with previous reports (Bandecchi et al., 1992; Hofmann-Lehmann et al., 1995; Hosie et al., 2009; Knotek et al., 1999). As gingivostomatitis is rare in SPF cats experimentally infected with FIV, co-infections with other infectious agents, such as feline calicivirus, might have contributed to this syndrome in naturally infected cats (Reubel et al., 1994; Tenorio et al., 1991). It is perhaps relevant that severe acute on chronic upper respiratory tract disease was diagnosed in four cats from Group 2 at post-mortem. Other abnormalities previously attributed to FIV-induced dysregulation and impairment of immune surveillance include dermatitis, ocular disease, renal insufficiency, lower urinary tract infections and other opportunistic infections (Barlough et al., 1991; Reche et al., 2010). Although neurological abnormalities have been reported in natural and experimental, acute and chronic FIV infections (Abramo et al., 1995; Dow et al., 1990; Podell et al., 1997, 1999), neither behavioural nor motor abnormalities were observed in our study population.

The mortality rate of 63% in the FIV-positive cats from Group 2 was markedly higher than the FIV-positive cats from Group 1, in which only one cat died during the study period (6%). Post-mortem examinations identified several pathological changes in the FIV-infected cats. Although FIV infection is generally associated with immunosuppression, immune hyperstimulation is evident during the early stages of infection, including B cell activation, manifested as polyclonal hypergammaglobulinemia (Hopper et al., 1989; Takano et al., 2012), expansion of CD8+ T cells with an activated phenotype and subsequent CD8+ T lymphocytosis (Beatty et al., 1996; Willett et al., 1993). Lymph node follicular hyperplasia and dysplasia was documented post-mortem in this study, confirming the findings of other studies of acute and terminal stage disease (Matsumura et al., 1993; Shelton et al., 1989; Yamamoto et al., 1988). In addition, reactive changes in the bone marrow, observed in 56% of cats in this study, have been documented previously in HIV and FIV infections (Bain et al., 2009; Breuer et al., 1998; Geller et al., 1985).

Lymphoma was the most common condition diagnosed at post-mortem in Group 2 (9/16 cats; 56%), which raises questions regarding potential interactions between virus, host, environment, and other factors that could have resulted in a predisposition to neoplasia. FIV strain variability was examined, since the Memphis FIV strains (Group 2) might have had greater oncogenic potential than the strains infecting the Chicago cohort (Group 1). However, a comparison of full-length *env* gene and long terminal repeat sequences revealed no significant differences (unpublished observation). It is possible that the local cluster of lymphoma in Group 2 was associated with another infectious agent, such as FeLV, but this seemed unlikely since FIV/FeLV Snap test (IDEXX) results were negative for FeLV antigen. However, we cannot exclude the possibility of regressive FeLV infection (Hofmann-Lehmann et al., 2001), since PCR testing to detect integrated proviral DNA in bone marrow was not performed. Breed was unlikely to play a role, as the cats from Group 2 diagnosed with lymphoma and those from Group 1 were all classified as domestic shorthairs and came from a variety of sources within their geographic area. The shelter environment in which the cats from Group 2 lived was likely to play a role in the development of the various clinical manifestations of FIV, although exposure to potential carcinogens cannot be ruled out.

Marked weight loss between enrolment and terminal measurement was evident among the cats that died over the study period, whereas healthy FIV-infected cats maintained relatively stable bodyweights (Fig. 2). Although monitoring bodyweight regularly can be a valuable tool in the care of FIV-positive cats and appeared to have some prognostic value in our study of FIV-positive cats, declining bodyweight is a relatively non-specific finding, as it is not unusual to observe weight loss during the progression of various other diseases.

The hallmark of FIV infection is an inverted CD4:CD8 ratio, which, as demonstrated here and elsewhere (Hoffmann-Fezer et al., 1992; Novotney et al., 1990; Tompkins et al., 1991), is largely the consequence of a loss of CD4+ T cells. Here we provide further evidence that FIV-positive cats had significantly lower CD4:CD8 ratios than those for FIV-negative cats over the 22-month study period in each of the cohorts (Fig. 3). We also observed a statistically significant decline in the CD4:CD8 ratio between the time of enrolment and the terminal sampling in those cats that died over the study period (Fig. 5). However, there was not a significant difference in median CD4:CD8 ratio at enrolment when cats classified as either healthy or not healthy were compared (Fig. 4). Both groups of cats had relatively low CD4:CD8 ratios with median values less than 1, regardless of their health status. Indeed, the cat that had the lowest CD4+ T lymphocyte count (0.09 K/μL) and the lowest CD4:CD8 ratio (0.08) at enrolment was classified as healthy and is still alive. Similar observations have been reported previously in FIV (Novotney et al., 1990) and HIV infections (Levy, 1988), where seropositive individuals remained asymptomatic despite significant loss of CD4+ T lymphocytes. The variability in CD4:CD8 ratios among healthy and not healthy cats in this study population also limited their utility in the prediction of disease progression.

Extrapolating from HIV infection, the plasma viral load has been postulated as a potential prognostic indicator for FIV infection. A study focusing on disease progression in SPF cats experimentally infected with two strains of FIV of different virulence reported a correlation between disease progression and increased plasma viral load (Diehl and Hoover, 1995). Similarly, the relationship between plasma viral load, disease progression and survival time was found in a study of naturally infected cats in Japan (Goto et al., 2002). In the present study, the median FIV load at the time of enrolment from Group 2 was 67-fold higher than that from Group 1 (*P* < 0.0001) and there was a marked difference in health status and mortality rate between groups over the following 22 months. It is not possible to discern whether an increased viral load was a cause or a consequence of poor health status, since that would have required knowledge of viral load kinetics and changes in health status from the time of initial infection in the FIV-infected ‘healthy’ and ‘not healthy’ cats; in this prospective study the data were collected from the time of enrolment. However, statistically significant differences in FIV load between cats from Group 2 classified as ‘healthy’ and ‘not healthy’ and cats that remained alive or died during the study were not apparent. Observation of these parameters over longer periods of time, preferably with larger group sizes, would be required to fully validate their utility in predicting disease progression.

It has been hypothesized that FIV clade B viruses are evolutionarily older and more host-adapted, hence less pathogenic, than those of clade A (Sodora et al., 1994). One possible explanation for the high morbidity and mortality in Group 2 is that those cats were infected with highly virulent strains of FIV. However, this hypothesis was not supported by the phylogenetic analyses performed, as cats from both groups were infected with comparable numbers of clade B and recombinant A/B viruses. Furthermore, sequences isolated from serial 6-monthly blood collections demonstrated that highly monophyletic groups were formed in 96% of the cats from Group 2, suggesting that viral isolates were not transmitted between cats despite unrestricted cohabitation (Bęczkowski et al., 2014). Although we cannot exclude the possibility that cats from Memphis were infected with more virulent FIV isolates, based on thorough phylogenetic analyses of full-length *env* sequences, this seems unlikely.

A limitation of this study is the restriction of enrolments to two distinct geographic areas; the inclusion of cats from a wider range of locations could have increased the variability in virulence of the wild type FIVs examined and their possible contribution to the onset of disease. Additionally, for the purposes of this study of naturally infected cats either adopted from shelters or rescued, it was necessary to estimate the ages of the cats and to use the date that the cat first tested FIV-positive since the precise dates of infection were not known. It has been reported previously that age at time of first exposure to FIV can influence the course of infection (George et al., 1993) and can determine the outcome of disease (Akhtardanesh et al., 2010). From a biological and behavioural perspective, the age of approximately 1–2 years old at the time of first diagnosis reported in this study is consistent with young cats engaging in territorial fights, especially if they are entire. The cats in our study were originally adopted from shelters and many were entire and had outdoor access at the time of shelter intake, both known lifestyle risk factors for FIV (Levy et al., 2006). In our study population, infection was more common in male cats, confirming the results of previous studies (Grindem et al., 1989; Hosie et al., 1989; Ishida et al., 1989; Levy et al., 2006; Yamamoto et al., 1989). Additionally, FIV status and multi-cat housing status could have affected the health of the cats in our study; it was not possible to discern whether they acted independently or as cofactors. FIV-negative cats matched to enrolled FIV-positive cats on housing status, as well as age and sex, were not available during the study enrolment period. While an extra layer of matching based on housing status would have been ideal, it was not achievable in this real-world study.

Based on the relatively large number of cats enrolled and the striking clinical differences observed between the two cohorts, we conclude that keeping FIV-infected cats in overcrowded conditions can have a significant impact on the risk of disease progression, particularly in cats which already have their immune systems compromised by FIV infection. In contrast, FIV-positive cats remained in relatively good health when living in stable, single cat households. Clinical signs of disease were not observed at 6-monthly physical examinations over a 24-month period of the cats from Group 1 that were housed in single cat households. The average duration of the ‘asymptomatic’ phase of infection remains unknown and could only be estimated following observations over a longer period. In this study, the selection of study participants from different locations and housing conditions minimized potential selection bias. It is apparent that the conclusions of this study would have been very different indeed had efforts been focused on only one of the cohorts presented herein.

## Conflict of interest

The authors declare that they have no competing interests.

## Figures and Tables

**Table 1:** Clinical abnormalities detected on physical examination in FIV-positive cats (*n* = 44) over the 22-month study period.

Description	Number of cats with clinical abnormalities (%)		
	Group 1 (*n* = 17)	Group 2 (*n* = 27)	All FIV-positive cats (*n* = 44)
Dental/oral cavity (faucitis)	12 (71)	19 (70)	31 (70)
Skin (alopecia, dermatitis)	10 (59)	12 (44)	22 (50)
Ears	3 (18)	4 (15)	7 (16)
Ocular disease	2 (12)	5 (19)	7 (16)
Cardiovascular	3 (18)	2 (7)	5 (11)
Digestive tract	0 (0)	4 (15)	4 (9)
Respiratory	0 (0)	2 (7)	2 (5)
Lymph nodes	0 (0)	0 (0)	0 (0)
Urogenital	0 (0)	0 (0)	0 (0)
Neurologic	0 (0)	0 (0)	0 (0)
Musculoskeletal	0 (0)	0 (0)	0 (0)
Weight loss/anorexia	0 (0)	22 (81)	22 (50)

**Table 2:** Breed, gender, age and age at first known positive feline immunodeficiency virus (FIV) ELISA test at enrolment.

	Group 1 (*n* = 17)	Group 2 (*n* = 27)	All FIV-positive cats (*n* = 44)
Breed	DSH (94%), DLH (6%)	DSH (74%), DLH (15%), Siamese X (11%)	DSH (82%), DLH (11%), Siamese X (7%)
Gender	FS (18%), MN (82%)	FS (33.3%), MN (66.6%)	FS (27%), MN (73%)
Age (years; median/range)	4/1–9	5/2–10	4.5/1–10
Age at first known positive FIV ELISA test (years; median/range)	2.3/0.2–7.8	1.6/0–4.6	1.8/0–7.8
Time from first known positive FIV ELISA test to enrolment (years; median/range)	1.3/0–3.3	3/0.1–8	2/0–8

DSH, domestic shorthair; DLH, domestic longhair; X, cross; FS, female spayed; MN, male neutered.

**Table 3:** Health status on the day of enrolment and post-mortem diagnoses for FIV-positive cats deceased during the study (Group 1, *n* = 1; Group 2, *n* = 17).

Cat #	Health status at enrolment	Post-mortem diagnosis
M3	NH	Lymphoma – retrobulbar
M5	NH	Lymphoma – bone marrow
M10	H	Lymphoma – bone marrow and lymph nodes
M11	H	Lymphoma – multicentric
M14	NH	Lymphadenomegaly; bone marrow hyperplasia
M16	NH	Pulmonary congestion and oedema with focal bacterial bronchopneumonia
M25	H	Upper and lower bacterial respiratory infection, probably secondary to viral infection
M26	NH	Chronic focal granulomatous nematode larval pneumonia; bone marrow hyperplasia
M30	NH	Lymphoid depletion; Aelurostrongylus abstrusus pneumonia; left front leg suppurative dermocellulitis; bone marrow hyperplasia, with erythroid and myeloid hyperplasia
M31	NH	Lymphoma – bone marrow
M33	NH	No post-mortem performed
M41	NH	Lymphoma – bone marrow and lymph nodes
M44	NH	Emaciation/nephritis/pancreatitis
M46	H	Lymphoma – bone marrow
M48	NH	Lymphoma – bone marrow and lymph nodes
M49	H	Lymphoma – bone marrow
M50	H	Undetermined/emaciation
C2	H	Cardiac failure, hypertrophic cardiomyopathy

C, Chicago, Group 1; M, Memphis, Group 2; H, healthy on the day of enrolment (no abnormalities detected on a physical examination); NH, not healthy on the day of enrolment (at least one abnormality detected on a physical examination).

**Table 4:** Organs with neoplastic lymphocyte infiltrates among FIV-positive cats diagnosed with lymphoma at post-mortem.

Cat #	Bone marrow	Lymph node	Spleen	Kidney	Liver	Jejunum	Tongue	Trachea	Heart	Eye
M3										+
M5	+	+	+							
M10	+	+								
M11	+	+	+	+	+	+	+	+	+	
M31	+									
M41	+	+	+							
M46	+									
M48	+	+								
M49	+									

## Appendix A Supplementary data

Table S1 FIV load, CD4+ T cell count, CD8+ T cell count and CD4:CD8 at the time of enrolment for FIV-positive cats from Group 1 (*n* = 17) and Group 2 (*n* = 27).

Table S2 CD4+ T cell count (K/μL) for FIV-positive cats from Group 1 (*n* = 14) and Group 2 (*n* = 24).

Table S3 The final clade assignment of full length *env* sequences from FIV-positive cats from Group 1 (*n* = 16) and Group 2 (*n* = 27) following recombination testing.^a^
