# Supplementary material for: Contrasting clinical outcomes in two cohorts of cats naturally infected with feline immunodeficiency virus (FIV)
Source: Vet Microbiol. 2015 Mar 23;176(1-2):50–60. doi: 10.1016/j.vetmic.2014.12.023 (PMC4332694; doi:10.1016/j.vetmic.2014.12.023)
Supplement: Table S1 — FIV load, CD4+ T cell count, CD8+ T cell count and CD4:CD8 at the time of enrolment for FIV-positive cats from Group 1 (n = 17) and Group 2 (n = 27). [file mmc1.docx]

**Supplementary Table 1**

FIV load, CD4+ T cell count, CD8+ T cell count and CD4:CD8 at the time of enrolment for FIV-positive cats from Group 1 (*n*=17) and Group 2 (*n*=27)

| Cat | FIV load  (genomes/mL blood) | CD4+  T cells  (K/µL) | CD8+  T cells  (K/µL) | CD4:CD8  ratio | Cat | FIV load  (genomes/mL blood) | CD4+  T cells  (K/µL) | CD8+  T cells  (K/µL) | CD4:CD8  ratio |
| --- | --- | --- | --- | --- | --- | --- | --- | --- | --- |
| M1 | NA | 0.87 | 0.65 | 1.35 | M46 | 418671 | 0.15 | 0.18 | 0.86 |
| M2 | 56867 | 1.74 | 2.58 | 0.68 | M47 | 140562 | 0.29 | 0.70 | 0.41 |
| M3 | 13991 | 0.33 | 0.29 | 1.16 | M48 | 3792122 | 0.17 | 0.25 | 0.66 |
| M5 | 19225 | 0.56 | 0.44 | 1.28 | M49 | 1383178 | 0.41 | 0.52 | 0.80 |
| M8 | 24147 | 0.55 | 0.29 | 1.86 | M50 | 23489 | 1.38 | 1.14 | 1.22 |
| M10 | 7724 | 0.71 | 0.69 | 1.03 | C1 | NA | 0.70 | 0.75 | 0.93 |
| M11 | 1666782 | 0.98 | 1.49 | 0.66 | C2 | NA | 0.40 | 0.51 | 0.78 |
| M12 | 418671 | 0.70 | 0.88 | 0.80 | C4 | 673 | 0.09 | 1.07 | 0.08 |
| M14 | 418671 | 0.45 | 0.35 | 1.27 | C5 | 2911 | 0.79 | 1.38 | 0.58 |
| M15 | 43138 | 0.87 | 0.93 | 0.94 | C6 | 741 | 0.48 | 0.49 | 0.97 |
| M16 | 13423 | 0.35 | 0.41 | 0.84 | C7 | 1099 | 0.40 | 0.45 | 0.88 |
| M20 | 31395 | 1.50 | 1.67 | 0.89 | C8 | 395 | 0.50 | 0.43 | 1.15 |
| M25 | 64843 | 0.36 | 0.42 | 0.86 | C9 | 18796 | 0.63 | 3.78 | 0.17 |
| M26 | 176551 | 0.54 | 0.42 | 1.29 | C10 | 540 | 1.77 | 2.39 | 0.74 |
| M28 | 24483 | 1.23 | 0.93 | 1.32 | C11 | 18796 | 0.45 | 1.06 | 0.42 |
| M29 | 2540 | 1.48 | 0.66 | 2.24 | C13 | 2104 | 0.46 | 0.75 | 0.61 |
| M30 | 2703217 | 0.13 | 0.70 | 0.19 | C14 | 692 | 0.97 | 0.77 | 1.26 |
| M31 | 41387 | 0.80 | 0.94 | 0.86 | C15 | 767 | 0.92 | 1.94 | 0.48 |
| M32 | 16176 | 0.38 | 0.65 | 0.58 | C17 | 668 | 0.49 | 0.68 | 0.71 |
| M33 | 2629546 | 0.20 | 0.87 | 0.23 | C18 | 16946 | 0.73 | 0.96 | 0.75 |
| M41 | 14583 | 0.34 | 0.19 | 1.76 | C21 | 45 | 0.93 | 0.99 | 0.94 |
| M44 | 179007 | 1.24 | 0.89 | 1.39 | C22 | 43 | 1.55 | 1.85 | 0.83 |

C, Chicago, Group 1; M, Memphis, Group 2; NA, not available
