# Supplementary material for: Contrasting clinical outcomes in two cohorts of cats naturally infected with feline immunodeficiency virus (FIV)
Source: Vet Microbiol. 2015 Mar 23;176(1-2):50–60. doi: 10.1016/j.vetmic.2014.12.023 (PMC4332694; doi:10.1016/j.vetmic.2014.12.023)
Supplement: Table S2 — CD4+ T cell count (K/μL) for FIV-positive cats from Group 1 (n = 14) and Group 2 (n = 24). [file mmc2.docx]

**Supplementary Table 2**

CD4+ T cell count (K/µL) for FIV-positive cats from Group 1 (*n*=14) and Group 2 (*n*=24).

| Cat No. | CD4+ T cell count (K/µL) | | | | ΔCD | Cat No. | CD4+ T cell count (K/µL) | | | | ΔCD4 |
| --- | --- | --- | --- | --- | --- | --- | --- | --- | --- | --- | --- |
|  | Enrolment | 6 months | 12 months | 18 months | (K/µL) |  | Enrolment | 6 months | 12 months | 18 months | (K/µL) |
| M1 | 0.87 | 0.36 | 0.34 | 0.26 | -0.61 | M44 | 1.24 | D | D | D | N/A |
| M2 | 1.74 | 0.36 | 0.76 | 0.62 | -1.12 | M46 | 0.15 | 0.19 | 0.18 | 0.18 | 0.03 |
| M3 | 0.33 | D | D | D | N/A | M47 | 0.29 | 0.14 | 0.14 | 0.1 | -0.19 |
| M5 | 0.56 | 0.15 | 0.16 | D | -0.4 | M49 | 0.41 | 0.31 | 0.27 | 0.04 | -0.38 |
| M8 | 0.55 | 0.21 | 0.2 | 0.15 | -0.4 | M50 | 1.38 | 1.32 | D | D | -0.07 |
| M11 | 0.98 | 0.14 | 0.48 | D | -0.5 | C2 | 0.4 | 0.36 | D | NA | -0.03 |
| M14 | 0.45 | 0.29 | 0.15 | 0.14 | -0.3 | C4 | 0.09 | 0.34 | 0.33 | NA | 0.24 |
| M15 | 0.87 | 1.03 | 1.09 | 0.47 | -0.4 | C5 | 0.79 | 0.72 | 0.3 | NA | -0.49 |
| M16 | 0.35 | 0.37 | 0.36 | D | 0.01 | C6 | 0.48 | 0.35 | 0.57 | NA | 0.09 |
| M20 | 1.5 | 0.81 | NA | 1.25 | -0.25 | C7 | 0.4 | 0.26 | 0.4 | NA | 0 |
| M25 | 0.36 | 0.39 | 0.68 | 0.09 | -0.27 | C8 | 0.5 | 0.86 | 0.6 | NA | 0.1 |
| M26 | 0.54 | 0.14 | 0.13 | D | -0.41 | C9 | 0.63 | 0.35 | 0.18 | NA | -0.45 |
| M28 | 1.23 | 0.89 | 1.77 | 0.9 | -0.34 | C11 | 0.45 | 0.35 | 0.45 | NA | 0 |
| M29 | 1.48 | 0.62 | 0.93 | 0.62 | -0.86 | C13 | 0.46 | 0.28 | 0.27 | NA | -0.18 |
| M30 | 0.13 | 0.15 | 0.26 | 0.1 | -0.04 | C14 | 0.97 | 1.16 | 1.09 | NA | 0.12 |
| M31 | 0.8 | 0.88 | 0.26 | D | -0.55 | C17 | 0.49 | 0.55 | 0.28 | NA | -0.21 |
| M32 | 0.38 | 0.32 | 0.35 | 0.21 | -0.17 | C18 | NA | 0.73 | 0.75 | NA | 0.02 |
| M33 | 0.2 | D | D | D | N/A | C21 | NA | 0.93 | 0.57 | NA | -0.36 |
| M41 | 0.34 | 0.35 | 0.12 | D | -0.22 | C22 | NA | 1.55 | 0.79 | NA | -0.76 |

ΔCD4, Change in CD4+ T cell count between enrolment and last available specimen; C, Chicago, Group 1; M, Memphis, Group 2;

D, deceased; NA, not available
