# Supplementary material for: Contrasting clinical outcomes in two cohorts of cats naturally infected with feline immunodeficiency virus (FIV)
Source: Vet Microbiol. 2015 Mar 23;176(1-2):50–60. doi: 10.1016/j.vetmic.2014.12.023 (PMC4332694; doi:10.1016/j.vetmic.2014.12.023)
Supplement: Table S3 — The final clade assignment of full length env sequences from FIV-positive cats from Group 1 (n = 16) and Group 2 (n = 27) following recombination testing.a [file mmc3.docx]

**Supplementary Table 3**

The final clade assignment of full length *env* sequences from FIV-positive cats from Group 1 (*n*=16) and Group 2 (*n*=27) following recombination testing ^a^

| Cat | Clade | Cat | Clade | Cat | Clade |
| --- | --- | --- | --- | --- | --- |
| M1 | B | M29 | B | C7 | A/B |
| M2 | A/B | M30 | B | C8A^b^ | A/B |
| M3 | B | M31 | A/B | C8C^b^ | B |
| M5A^b^ | B | M32 | B | C9 | B |
| M5C^b^ | B | M33 | A/B | C10 | B |
| M8 | A/B | M41 | A/B/D | C11 | B |
| M10 | B | M44 | B | C13 | A/B |
| M11 | B | M46 | B | C14 | A/B |
| M12 | B | M47 | A/B | C15 | A/B |
| M14 | B | M48 | A/B | C17 | B |
| M15 | B | M49 | B | C18 | A/B |
| M16 | B | M50 | A/B | C21B^b^ | B |
| M20 | A/B | C2 | B | C21C^b^ | B/A |
| M25 | B | C4 | A/B | C22 | B |
| M26 | B | C5 | A/B |  |  |
| M28 | B | C6 | B |  |  |

C, Chicago, Group 1; M, Memphis, Group 2;

^a^ Genetic Algorithm Recombination Detection (GARD) method (Delport et al., 2010) and Recombination Detection Program (RDP; Martin et al., 2010).

^b^ Sequences from the same individual with previously identified non-monophyletic clustering, at different time points (Bęczkowski et al., 2014).
